# Clinical Patterns and Treatment Response of Patients With Mycosis Fungoides a Retrospective Study

**DOI:** 10.7759/cureus.21231

**Published:** 2022-01-14

**Authors:** Heba Y Alojail, Hamza Alshehri, Feroze Kaliyadan

**Affiliations:** 1 Dermatology, King Faisal University, Al Ahsa, SAU; 2 Dermatology, Aseer Central Hospital, Asser, SAU

**Keywords:** hyperpigmented mycosis fungoides, hypopigmented mycosis fungoides, narrow band-ultraviolet b (nb-uvb), cutaneous t-cell lymphoma, mycosis fungoides

## Abstract

Background

Mycosis fungoides (MF) is one of the primary cutaneous T-cell lymphomas and is considered to be the most common extranodal non-Hodgkin lymphomas. MF is characterized by different subtypes based on clinical presentation and immunophenotyping studies. We aimed to study the clinical patterns and treatment response in cases of MF among the patients attending a tertiary referral hospital in Saudi Arabia.

Methodology

A retrospective study, case record-based study was done to review all the patients diagnosed with MF from January 2011 to May 2016. All cases with histopathological confirmation and immunophenotyping were included in the study. Treatment follow-up was reviewed for 9 months in all cases. Treatment response was graded based on a global physician assessment-complete response, good response, moderate response, and minimal or no response.

Results

Out of 34 cases of MF included in the study, 11 were hyperpigmented MF, 21 were hypopigmented MF, and there was one case each of poikilodermatous MF and pagetoid reticulosis. Of the total, fourteen (66.7%) of hypopigmented MF patients showed a complete response to phototherapy Narrowband UVB (NB-UVB) in combination with topical corticosteroids. Nine (81.8%) of hyperpigmented MF patients showed partial to the phototherapy NB-UVB in combination with the topical corticosteroid. Among the other types; one case of poikilodermatous MF (2.9%) showed a moderate response to phototherapy NB-UVB with topical corticosteroid and systemic acitretin.

Conclusions

The most common type of MF seen in our study was the hypopigmented type, affecting a younger age group, and the same showed a good response to phototherapy NB-UVB combined with topical corticosteroids.

## Introduction

Mycosis fungoides (MF) is considered to be a subset of primary cutaneous T-cell lymphomas, which is regarded as one of the most common types of extranodal non-Hodgkin lymphomas. The pathogenesis of MF is believed to result from chronic antigenic stimulation that leads to uncontrolled clonal expansion and the accumulation of T cell helper memory cells in the skin [1]. MF, which is generally indolent in behavior, has different variants according to the World Health Organization and European Organization for Research and Treatment of Cancer classification. Cutaneous lymphomas represent 3.9% of all non-Hodgkin lymphomas, with MF comprising the majority of cases [2]. Mycosis fungoides is the most common, which represents about 70% of all cases where the patch and plaque stages are the most commonly seen in MF [3]. There are very few studies related to the prevalence of cutaneous lymphomas in Saudi Arabia. A single-center retrospective study by Binamer included 125 cases of which the commonest were the hypopigmented and poikilodermatous variants [3]. We hoped to build upon the study by Binamer and also aimed to study the treatment response in our group of patients.

## Materials and methods

**Figure 1:**
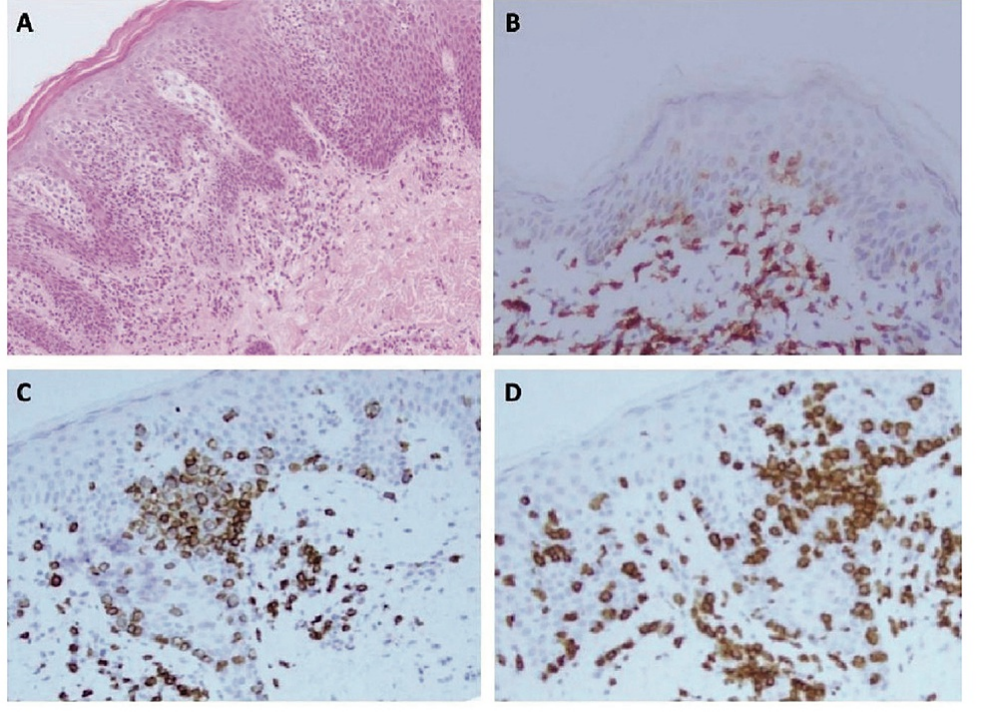
Histopathology and immunohistochemistry of Mycosis fungoidesb: A: Hematoxylin and eosin ; B: CD4; C: CD8; D: CD3.

We retrospectively reviewed all diagnosed cases of MF among patients who were diagnosed and followed up in our dermatology clinic from January 2011 to May 2016. All patients’ data were collected and classified according to various parameters like age, gender, skin type, and morphology of the lesions. All of them had undergone skin biopsy procedures to confirm the diagnosis, and the immunohistochemistry was done using CD3, CD4, and CD8 to identify the type of MF. The hypopigmented MF was stained with CD3+ and CD8; the hyperpigmented MF was stained with CD3+ and CD4+ (Figure 1), the poikilodermatous MF was stained with CD3+, CD8+, and weakly positivity of CD4, and pagetoid reticulosis was stained with CD3+, CD4+, CD8+, and CD30+. The assessment of the response to treatment was based on a global assessment by the physician, taking into account clinical and histological follow-up for all patients included in the study. All patients were categorized into three groups; Group 1 included patients treated with narrowband UVB (NB-UVB) alone, Group 2 was treated with NB-UVB in combination with a topical steroid, Group 3 was treated with NB-UVB in combination with topical steroid and acitretin. As far as the topical steroid used was concerned, all were initially started on Clobetasol cream 0.05%, applied twice daily for three months then shifted to Betamethasone valerate 1% applied twice daily for a further three to six months. All patients were followed up initially at the end of one month, followed by repeat visits at the end of the third month and ninth month.

## Results

**Figure 2:**
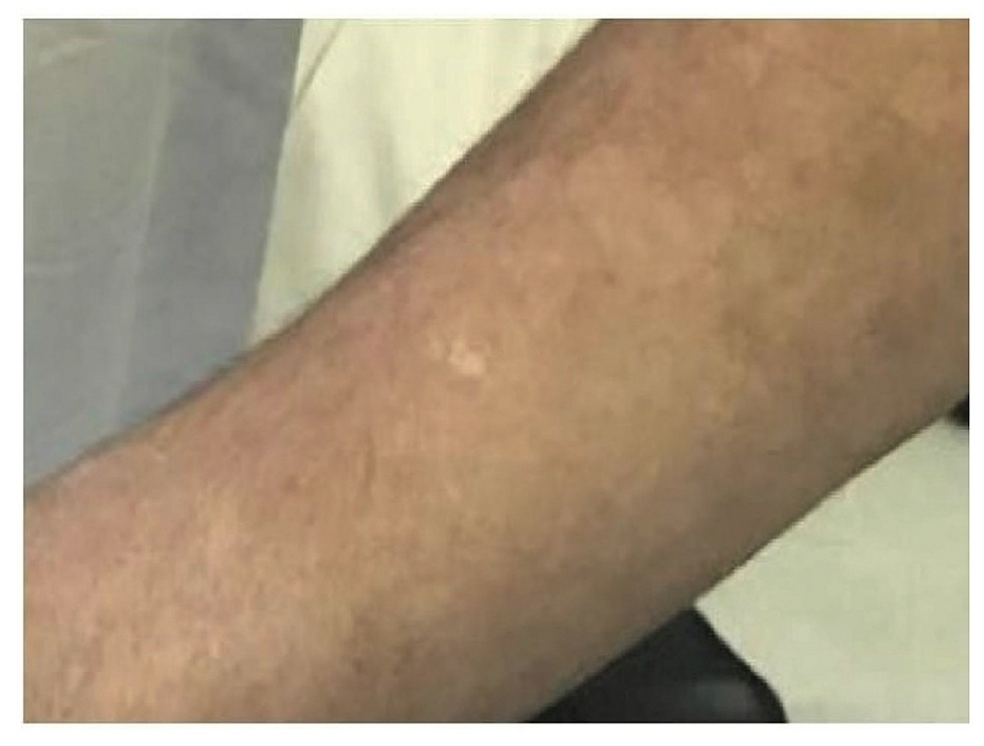
Hypopigmented Mycosis Fungoides

The general characteristics of the patients are given in Table 1 and Figure 2. According to the age-group category, it was noticed that all patients < 20 years had the hypopigmented type of MF [n = 13 (38.2%)], while the patients (20-39 years) had hypopigmented MF [n = 7 (24.2%)] more than hyperpigmented MF (n = 5 (14.7%)]. Still, the patients > 40 years commonly had the hyperpigmented MF [n = 6 (17.6%)], one patient had the hypopigmented MF (2.9%), one patient had the poikilodermatous MF (2.9%), and one patient had the pagetoid reticulosis variant of MF (2.9%). The remaining patients were excluded because of poor compliance.

**Table 1:** General characteristics of the study participants. MF: Mycosis Fungoides

Variables	(%)
Gender (n=34)	
Male	25 (75.8%)
Female	8 (24.2%)
Age (years) (n=34)	
0-9	6 (17.6%)
10-19	7 (20.6%)
20-29	4 (11.8%)
30-39	7 (20.6%)
40 and above	10 (29.4%)
Type of MF (n=34)	
Hyperpigmented MF	11 (32.4%)
Hypopigmented MF	21 (61.8%)
Poikilodermatous MF	1 (2.9%)
Pagetoid retticulosis	1 (2.9%)
CD	
CD3	34 (100%)
CD4	12 (35.3%)
CD8	23 (67.6%)

NB-UVB alone

In the first three months, only one patient (4.8%) with a hypopigmented MF had an excellent response to NB-UVB alone with a complete clearance (the patient was < 10 years of the age and had a minimal number of hypopigmented MF lesions), while five patients of a hypopigmented MF (23.8%) had a good response during the six months of the treatment where two patients were < 10 years and three patients were 1019 years of their ages (Table 2).

NB-UVB + topical corticosteroids

During the first six months, 14 patients of hypopigmented MF (66.7%) three patients < 10 years and 11 patients [n = 7 (age: 10-19 years), n = 4 (age: 20-29 years)], while one hypopigmented MF patient (4.8%) and one hyperpigmented MF patient (9.9%) had complete clearance during the nine months. Of the hyperpigmented MF patients, [n = 9 (81.8%)] responded well to the combined therapy of NB-UVB and topical corticosteroids (Table 2). Efficacy of NB-UVB + topical corticosteroids + acitretin: Only one patient underwent this regimen, a case of poikilodermatous MF that showed a good response at the end of nine months (Table 2).

**Table 2:** Response to the treatment according to the MF type. NB-UVB: Narrow-band ultraviolet B, M: months

Final DX of MF	NB-UVB alone						NB-UVB+Topical corticosteroids						NB-UVB+acitretin					
	1-3 M		3-6 M		6-9 M		1-3 M		3-6 M		6-9 M		1-3 M		3-6 M		6-9 M	
	N	%	N	%	N	%	N	%	N	%	N	%	N	%	N	%	N	%
Hyperpigmented (N=11)	0	0	0	0	5	45.4	0	0	3	27.3	1	9.1	0	0	0	0	2	18.2
Hypopigmented (N=21)	3	4.8	12	57.1	1	3.2	4	19.04	1	4.8	0	0	0	0	0	0	0	0
Poikilodermatous MF (N=1)	0	0	0	0	0	0	0	0	0	0	0	0	0	0	0	0	1	100

## Discussion

A previous study has found that cutaneous T-cell lymphoma was the most common type of skin cancer in our geographical area [4]. Clinicopathological correlation in this study found that the hypopigmented MF was the most common type in childhood and the youth, especially in skin type IV. At the same time, the hyperpigmented MF was more common after 30 years of age [5]. Ultraviolet light-based therapy; specifically, ultraviolet B (UVB) light phototherapy and psoralen plus ultraviolet A light (PUVA) photochemotherapy have been a mainstay of treatment of MF for the past 50 years. Initially, it was used exclusively as monotherapy, but more recently it has been commonly used as part of a multimodality therapeutic regimen. Its efficacy is beyond dispute, but how to best harness this while minimizing side effects has yet to be fully determined [1]. Narrowband UVB (NB-UVB; 311 nm) is used more frequently than PUVA in early-stage MF because of its similar efficacy; there are also increased rates of skin cancer with PUVA [6].

During follow-up of all the patients treated with a different modality of treatment, it was noticed that the hypopigmented MF responded well to the phototherapy of NB-UVB alone and showed more rapid response if it was combined with the topical corticosteroid during the first three months of the initiation of therapy. This is similar to other studies. Otherwise, the hyperpigmented MF usually shows a good response after six months if the NB-UVB is combined with a topical corticosteroid. The exact mechanism of the therapeutic efficacy of NB-UVB is not fully understood, one of the mechanisms is the apoptosis induction for the lymphocytes where they seem to be more sensitive to NB-UVB compared to other cells like keratinocytes [7], another theoretical mechanism has UVB did not affect CD4 and CD8 expression or their ratio. In contrast, the expression of IL-2R (CD25) was only slightly reduced, leading to IL-2 reduction, whereas IL-2 is considered as a T-cell growth factor that affects T cell proliferation and survival [8]. Regarding the absorption of UVB, UVB is mainly absorbed by epidermal components, including keratinocytes, melanin, and Langerhans cells [9,10]. The mechanism of action of topical steroids in MF includes IL-2R modulation to reduce the production of IL-2 [11]. The explanation for relatively better improvement for the hypopigmented MF with combined therapy of the NB-UVB and topical steroid is attributed to the synergic effects on IL-2R (CD25). Hyperpigmented MF is associated with an increased amount of melanin. It is postulated that NB-UVB photons will be absorbed by the melanin which in turn leads to the hyperpigmented skin lesions not getting an optimal therapeutic dose of NB-UVB, thereby leading to a slower response [1,7,12,13]. The small sample size, the global assessment of treatment response, and the retrospective design are the main limitations of the study.

## Conclusions

The most common type of MF seen in our series was the hypopigmented type, affecting a younger age group, and the same showed a good response to phototherapy (NBUVB) combined with topical corticosteroids.
